# Supplementary material for: Cancer-Specific Immune Prognostic Signature in Solid Tumors and Its Relation to Immune Checkpoint Therapies
Source: Cancers (Basel). 2020 Sep 1;12(9):2476. doi: 10.3390/cancers12092476 (PMC7563367; doi:10.3390/cancers12092476)
Supplement: Supplementary file 1 [file cancers-12-02476-s001.pdf]

Supplementary Materials: Cancer-Specific Immune Prognostic Signature in Solid Tumors and Its Relation to Immune Checkpoint Therapies

Shaoli Das, Kevin Camphausen and Uma Shankavaram

Supplementary Figures

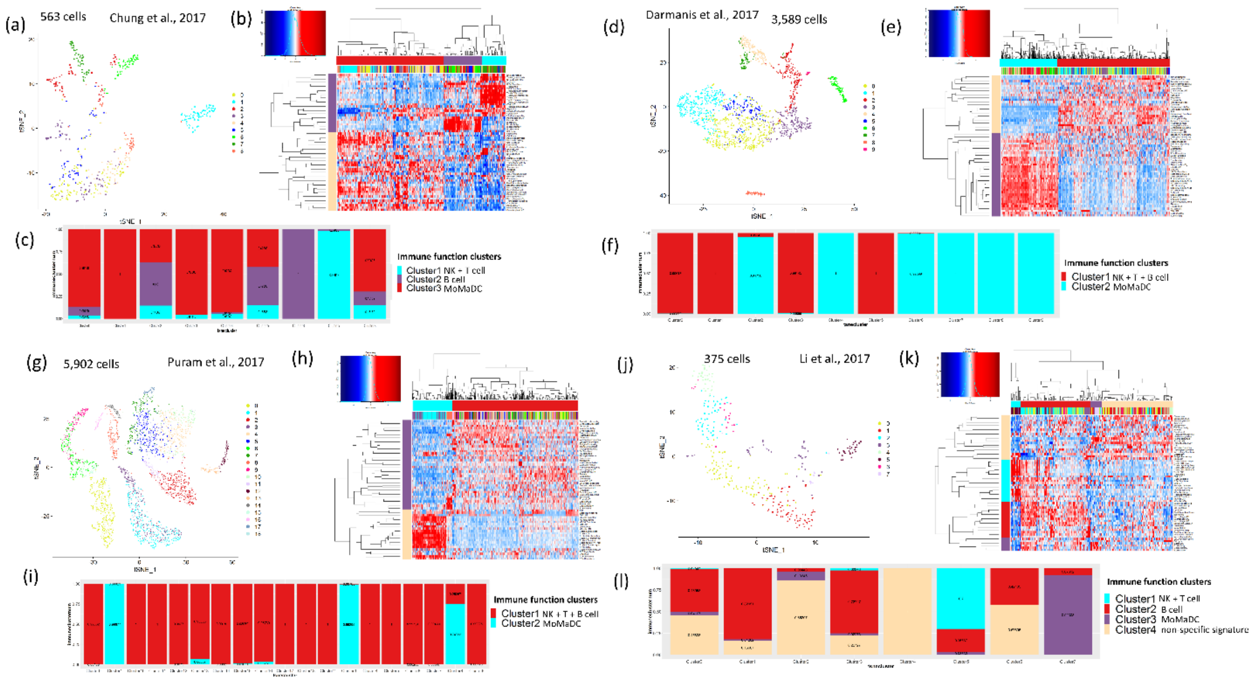

Figure S1. ssGSEA of scRNA-seq data of tumor samples from four solid tumor histologies (breast, colon, glioblastoma, and head and neck tumors) identifies three or two dominant immune-function-associated cell clusters. One of these clusters is enriched for monocytes, macrophages, dendritic cells (MoMaDC), another cluster is enriched for NK-, T-cells, and another is enriched for B-cell functions. For head and neck tumors and glioblastomas, B-cell enrichments does not form a distinct cluster but forms a unified cluster along with NK- and T-cells. (a) Analysis of a breast cancer scRNA-seq data set identifies nine cell clusters from unsupervised t-SNE clustering. (b) These clusters were assigned to three distinct sets of immune functions using ssGSEA, as seen from the heat map (one related to monocytes, macrophages, TLR and dendritic cells; one related to B-cell functions; and the other related to NK- and T-cell functions). (c) The stacked bar plot shows that cell clusters 1, 3, and 4 are enriched for functions related to monocytes, macrophages, and dendritic cells; the cell cluster 6 is enriched for B cell functions; and cell cluster 7 is enriched for NK- and T- related functions. (d) Analysis of a GBM scRNA-seq data set identifies 10 cell clusters from unsupervised t-SNE clustering. (e) These clusters were assigned to two distinct sets of immune functions using ssGSEA, as seen from the heat map (one related to monocytes, macrophages, TLR, dendritic cells and the other related to NK-, T- and B-cell functions). (f) The stacked bar plot shows that cell clusters 0, 1, 3, and 5 are enriched for functions related to monocytes, macrophages and dendritic cell and that cell clusters 2, 4, 6, 7, 8, and 9 are enriched for NK-, T- and B-cell related functions. (g) Analysis of a GBM scRNA-seq data set identifies 19 cell clusters from unsupervised t-SNE clustering. (h) These clusters were assigned to two distinct sets of immune functions using ssGSEA, as seen from the heat map (one related to monocytes, macrophages, TLR and dendritic cells and the other related to NK-, T- and B-cell functions). (i) The stacked bar plot shows that cell clusters 0, 2, 4, 5, 6, 7, 9, 10, 11, 12, 13, 14, 15, 16, 17, and 18 are enriched for functions related to monocytes, macrophages and dendritic cells and that cell clusters 1 and 3 are enriched for functions related to NK-, T- and B-cells. (j) Analysis of a colon cancer scRNA-seq data set

identifies eight cell clusters from unsupervised t-SNE clustering. (k) These clusters were assigned to four distinct sets of immune functions using ssGSEA, as seen from the heat map (one related to monocytes, macrophages, and dendritic cells; one related to B-cell functions; one related to NK- and T-cell functions; and one non-specific cluster). (l) The stacked bar plot shows that cell clusters 1, and 3 are enriched for functions related to monocytes, macrophages, and dendritic cells; the cell cluster 5 is enriched for NK- and T-cell functions; and cell cluster 7 is enriched for B-cell functions.

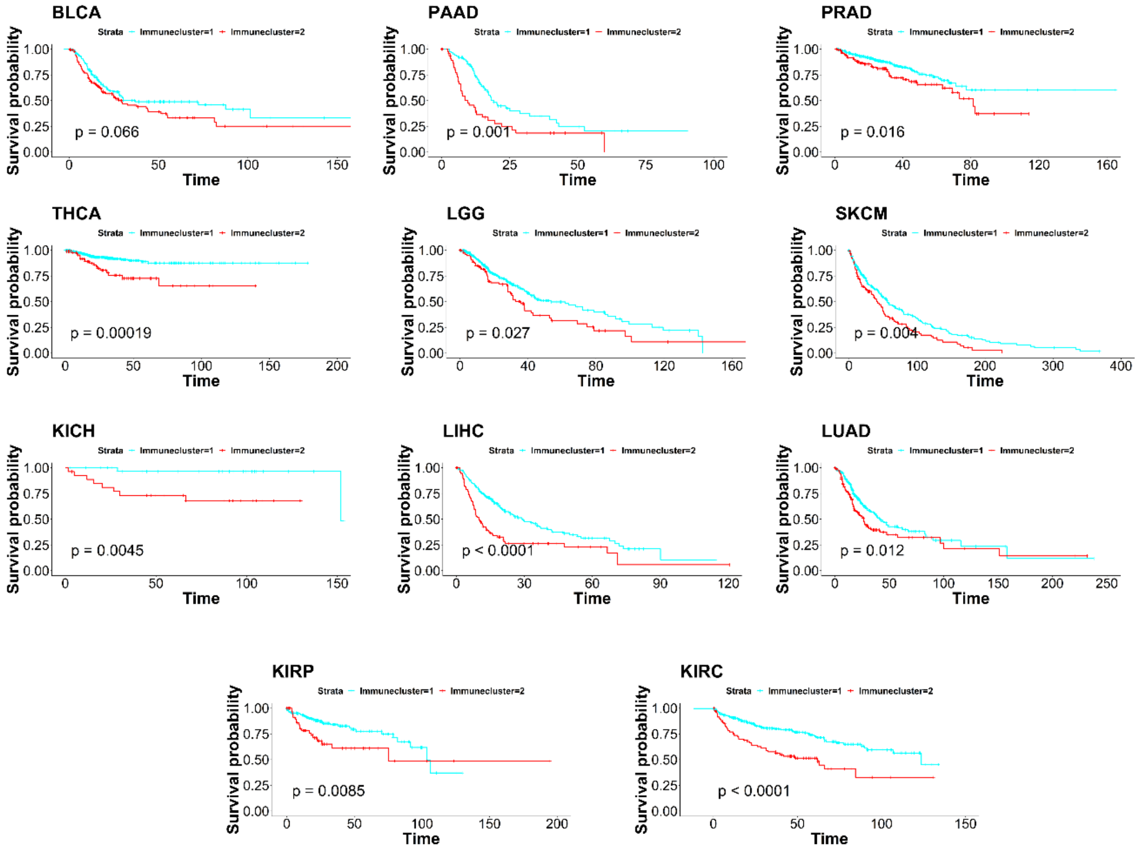

Figure S2. Kaplan-Meier analysis of cancer samples between the two sample clusters from the pan-cancer expression analysis of 155 immune signature genes (from Figure 3B) shows that in most cancer types (LGG, SKCM, LUAD, KIRC, KIRP, KICH, PRAD, PAAD, LIHC, THCA, and BLCA), cluster 1 (overexpression of bad prognosis genes) is significantly associated with poor disease-free survival.

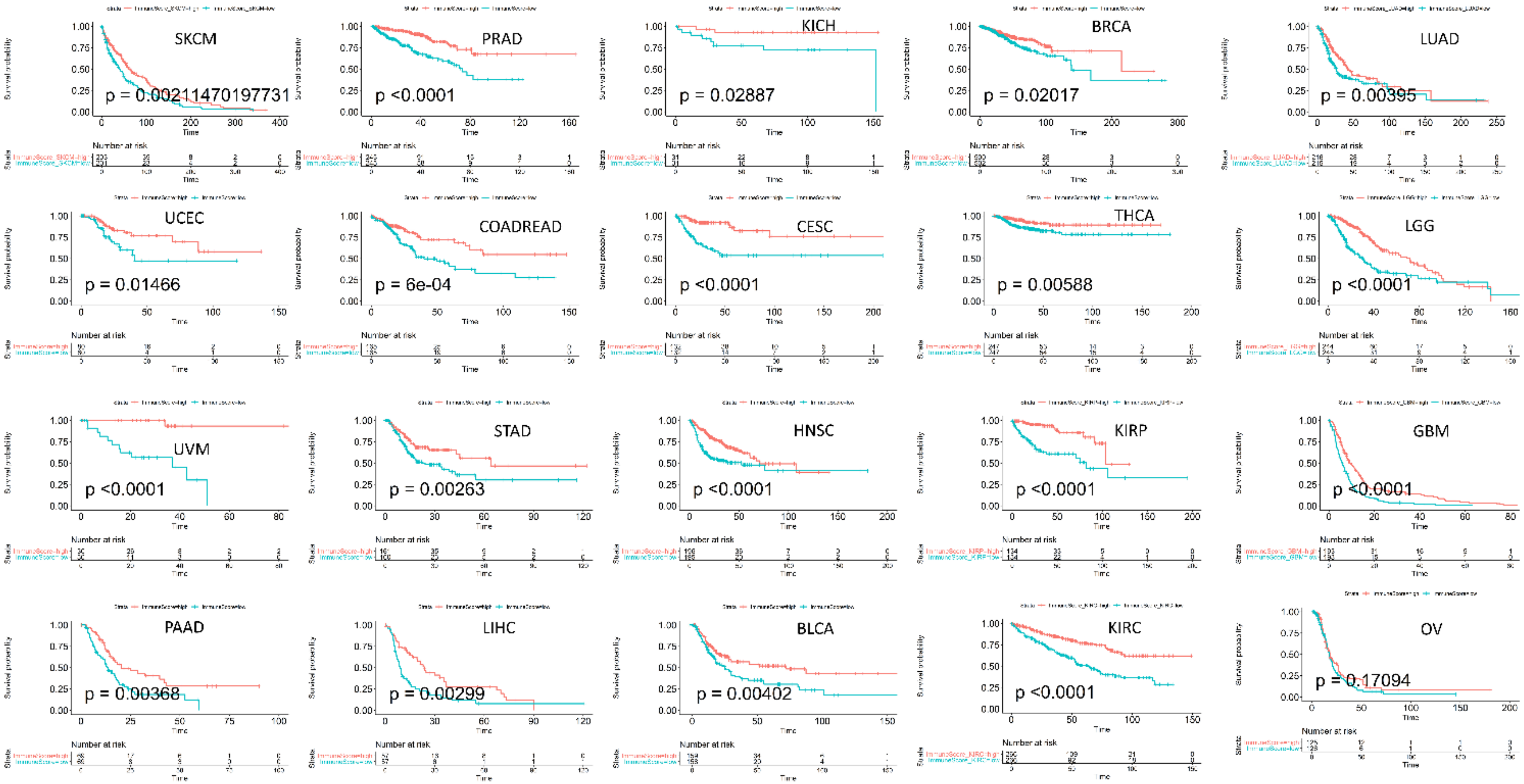

Figure S3. Kaplan-Meier analysis of disease-free survival of cancer samples stratified by high (>median, red line) or low (<median, blue line) immune scores specific for each of the 20 cancer types shows that high immune scores are significantly associated with better disease-free survival in all cancer types.

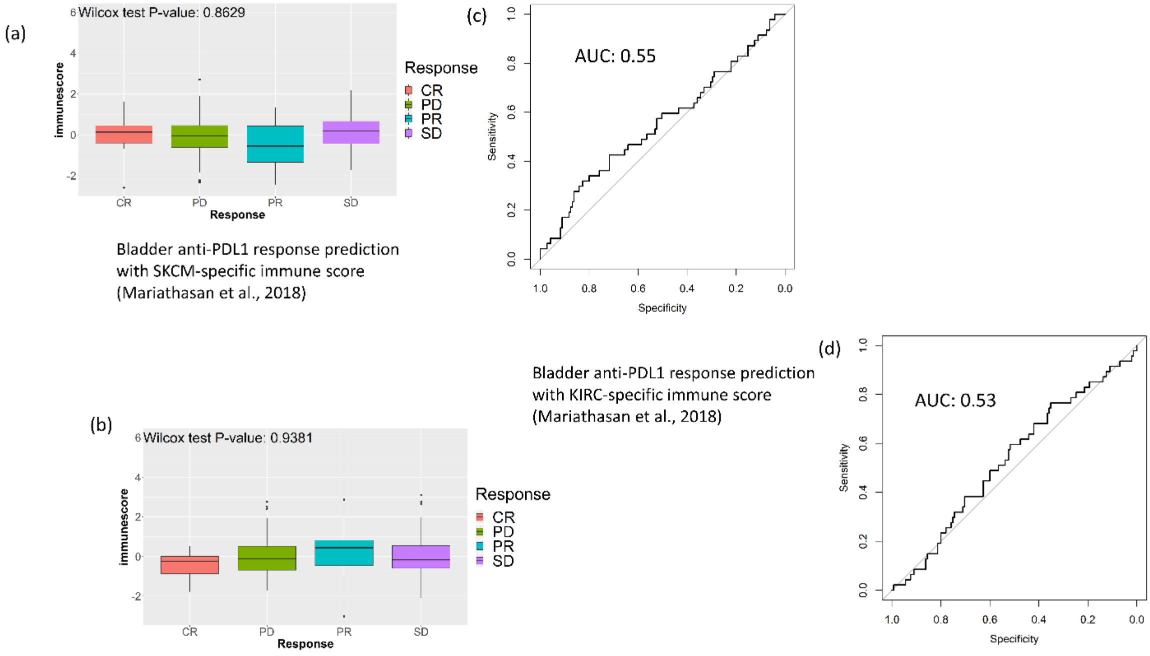

Figure S4. Immune score models for nonspecific cancer types could not predict response to checkpoint blockade therapy in bladder cancer (Mariathasan et al., 2018). (a–b) Immune score specific to SKCM (a) and KIRC (b) was not higher in tumors that responded (complete or partial response, CR/PR) compared to tumors that did not responded (standard or progressive disease, SD/PD) in anti-PDL1-treated bladder cancer patients. (c–d) Immune score specific to SKCM (c) and KIRC (d) could not predict objective response to anti-PDL1 treatment in bladder cancer patients. The AUC for predicting responsive tumors (CR/PR) versus non-responsive tumors (SD/PD) was close to that of random prediction (0.5).

Table S1. All genes associated with disease-free survival in 20 TCGA cancer types; chosen using elastic net and Kaplan-Meier analysis.

Gene	Cancer	Coeff
RPL31	GBM	0.010127
RPS16	GBM	0.007807
EIF3G	GBM	0.021535
PLOD2	GBM	-0.02265
FRAT2	GBM	0.220046
MYD88	GBM	-0.02697
TNFSF4	GBM	-0.10415
VNN1	GBM	-0.01664
SLC12A8	GBM	-0.03837
SNX10	GBM	-0.06819
CHI3L1	GBM	-0.03997
BCL2A1	GBM	-0.24106
CFLAR	GBM	-0.04284
BANK1	LGG	-0.04219
BCL7A	LGG	0.05836
CAPG	LGG	-0.02874
CASP3	LGG	-0.03997
CEACAM1	LGG	-0.04833
CHST15	LGG	0.03857

EIF3H	LGG	0.018127
TMEM255A	LGG	-0.02755
FBXO6	LGG	-0.01493
GIMAP2	LGG	-0.00525
HESX1	LGG	-0.017
HSH2D	LGG	-0.02217
IL22RA1	LGG	-0.03903
NPL	LGG	-0.05264
PARP10	LGG	-0.00056
PSEN2	LGG	-0.00546
PSMB8	LGG	-0.00104
RAG1	LGG	-0.02385
RPL3	LGG	0.002712
RPL7	LGG	0.004706
SOCS1	LGG	-0.02082
SPATS2L	LGG	-0.02545
TNFAIP6	LGG	-0.01694
TNFRSF12A	LGG	-0.0436
TRIM6	LGG	-0.0133
BIRC5	SKCM	0.060904
CD7	SKCM	-0.15728
CDC6	SKCM	0.012193
CDCA2	SKCM	-0.05299
DDX58	SKCM	-0.44534
DDX60	SKCM	0.590045
DEPDC1	SKCM	-0.54929
DONSON	SKCM	0.113839
DTL	SKCM	-0.15809
ECT2	SKCM	-0.25612
GBP4	SKCM	0.056467
GINS2	SKCM	-0.69122
IFITM1	SKCM	0.229638
IL12A	SKCM	0.044932
IL12RB2	SKCM	-0.18564
IL18RAP	SKCM	0.552766
IRF1	SKCM	-0.01035
IRF7	SKCM	0.380559
KLRC1	SKCM	0.311587
KLRD1	SKCM	0.141009
LAP3	SKCM	0.013529
MKI67	SKCM	0.536968
PARP12	SKCM	0.131166
PYHIN1	SKCM	0.289861
RACGAP1	SKCM	-0.33746
SKA1	SKCM	-0.94405
STAT1	SKCM	0.061859
TLR2	SKCM	0.132438
TLR8	SKCM	0.115879
UBE2C	SKCM	0.036744
CDCA2	LUAD	-0.02714

DEPDC1B	LUAD	-0.11074
DLGAP5	LUAD	-0.06315
DTL	LUAD	-0.01635
ECT2	LUAD	-0.08089
KIF14	LUAD	-0.02378
KIF20A	LUAD	-0.04956
MAD2L1	LUAD	0.011961
SHCBP1	LUAD	-0.00519
SLC12A8	KIRC	-0.40187
AURKA	KIRP	-0.0557
AURKB	KIRP	-0.03724
BUB1	KIRP	-0.01275
CDC20	KIRP	-0.06612
CDCA8	KIRP	-0.02403
CDK1	KIRP	-0.00902
CDKN3	KIRP	-0.00877
CENPA	KIRP	-0.02973
CENPF	KIRP	-0.03067
DEPDC1	KIRP	-0.0431
EIF3E	KIRP	-0.02167
EIF3H	KIRP	-0.04278
FOXM1	KIRP	-0.01895
HJURP	KIRP	-0.04352
HMMR	KIRP	-0.01766
KIF11	KIRP	-0.00188
KIF18B	KIRP	-0.04813
KIF20A	KIRP	-0.01681
MKI67	KIRP	-0.00906
NCAPG2	KIRP	-0.00523
NDC80	KIRP	-0.0141
NUF2	KIRP	-0.01939
PLK4	KIRP	-0.03815
PTTG1	KIRP	-0.0045
RPL27	KIRP	-0.03856
RPL30	KIRP	-0.00685
RPL35A	KIRP	-0.04144
SERPING1	KIRP	0.190392
SPC25	KIRP	-0.07121
TOP2A	KIRP	-0.00297
TPX2	KIRP	-0.00618
TRIP13	KIRP	-0.01367
UBE2C	KIRP	-0.03484
C1S	LIHC	0.027476
IRF2	LIHC	0.005467
MBOAT7	LIHC	-0.01116
MCM10	LIHC	-0.00126
MKI67	LIHC	-0.01374
RACGAP1	LIHC	-0.01617
RASGRP3	LIHC	0.001385
REPS2	LIHC	0.096182

RRP12	LIHC	-0.07219
STMN1	LIHC	-0.02358
BCL2	KICH	0.16179
EAF2	KICH	0.026256
BANK1	UVM	-0.50914
CDKN3	UVM	-0.34223
CENPA	UVM	-0.02007
RPL29	UVM	0.034258
RPS10	UVM	0.010025
RPS18	UVM	0.018559
RPS28	UVM	0.235403
RRM2	UVM	-0.00156
SEC11C	UVM	-0.5662
SPATS2	UVM	-0.01274
KIR3DL1	HNSC	0.061815
NFATC3	HNSC	0.051518
RASSF5	HNSC	0.047304
STAT3	HNSC	0.006566
STX3	HNSC	-0.04074
TAL1	HNSC	0.024819
TNFRSF12A	HNSC	-0.02452
CDR2	CESC	-0.06467
FAM129C	CESC	0.103777
FRK	CESC	0.031133
TCL1A	CESC	0.140501
TNFAIP6	CESC	-0.04312
VCAN	CESC	-0.13779
GUCY1B3	UCEC	-0.13335
SMPD3	UCEC	0.055162
MAD2L1	THCA	-0.01356
CD69	BRCA	0.123017
CTLA4	OV	0.042074
MZB1	OV	0.17865
SOCS1	OV	-0.06774
TNFRSF17	OV	0.009313
ALOX12	PRAD	-0.0046
ASAP2	PRAD	0.022714
ASGR1	PRAD	-0.41033
BIRC5	PRAD	-0.03426
CD38	PRAD	0.095152
GMNN	PRAD	-0.15365
MAD2L1	PRAD	-0.00822
PTTG1	PRAD	-0.03482
SPOCK2	PRAD	0.000269
STMN1	PRAD	-0.094
ZWINT	PRAD	-0.0485
CD44	PAAD	-0.32402
CDKN3	PAAD	-0.20091
CEP55	PAAD	-0.04648
ETS1	PAAD	-0.20664

KIF20A	PAAD	-0.49693
TPX2	PAAD	-0.48558
TTK	PAAD	-0.46591
UBE2C	PAAD	-0.01529
RCAN3	COADREAD	0.016729
SOCS1	COADREAD	0.111016
SPP1	COADREAD	-0.06986
VEGFA	COADREAD	-0.0339
CDCA7	STAD	0.002116
E2F8	STAD	0.027942
MELK	STAD	0.02538
NCAPG2	STAD	0.011524
STON2	STAD	-0.09419
TNFSF4	STAD	-0.05426
SIRPG	BLCA	0.050077

© 2020 by the authors. Licensee MDPI, Basel, Switzerland. This article is an open access article distributed under the terms and conditions of the Creative Commons Attribution (CC BY) license (http://creativecommons.org/licenses/by/4.0/).
